# Our Institution

**Published:** 1837

**Authors:** 


### III.--ORIGINAL.

### THE WESTERN JOURNAL.

*E Sylvis Nuncius.*

CINCINNATI, OHIO, OCTOBER 1, 1837.

### Art. I.—Our Institution.

We are happy to announce to our medical friends, that we shall be well prepared, (excepting in ideas, which, however, are considered *immaterial*) for the re-opening of our College at the appointed time. The edifice will be much improved in its condition from what it was last autumn, the hospital better organized, and our physical means of instruction, much enlarged. We have just received (Sept. 13) an invoice of nine boxes of books, chemical apparatus, anatomical preparations—both healthy and morbid—and surgical and obstetrical instruments and *appareil*, purchased, by Prof. Parker, in Paris. They were shipped from Havre, on the first of July. At that time, the professor went to London—*carpere colligere*—and is now on the return voyage, with his additional acquisitions. Our colleague, Prof. Rogers, who, although a physician, does not practice medicine, is sharpening his senses on the rocks of Virginia, being one of the Geologists of that State; but will meet his Chemical apparatus in New-York, and be with it in his laboratory, before the opening of the session. The first introductory lecture will be delivered, on Saturday night the 29th of October, in the great Hall of the College Edifice, which has been beautifully refitted; and, during the week that follows, several of the professors will deliver didactic as well as introductory lectures. It would be well, therefore, for such as may be able, among the students, who intend to enter our institution, to be in *before* the last Saturday of October. It is the earnest desire of the Faculty, to give extended courses of instruction; and they cannot but hope, that our students of medicine will encourage them in so laudable a purpose, by an early and a protracted attendance. By the laws of the institution, they may, it is true, become, *technically*, qualified for graduation, if they do not arrive till the 20th of November; but the Professors have an ambition, which cannot be gratified by numbers alone—*the ambition to make good physicians, surgeons, and obstetricians*—and short sessions are little in favor of such an object. The qualifications for a degree are expected to be proportionate to the opportunities created by the institution. If the students *neglect* to avail themselves of the *whole*, and should happen to be *pumped dry*, within a few minutes after they enter the *green room*, it will not be because the pump has too great a *lift*, but that the fountain is too *shallow*. D.

